# Detection of estrogen receptors ER-alpha and ER-beta in human ejaculated immature spermatozoa with excess residual cytoplasm

**DOI:** 10.1186/1477-7827-4-36

**Published:** 2006-07-17

**Authors:** Vittoria Rago, Laura Siciliano, Saveria Aquila, Amalia Carpino

**Affiliations:** 1 Department of Cell Biology, Faculty of Pharmacy, University of Calabria, Cosenza, Italy; 2 Department of Pharmaco-Biology, Faculty of Pharmacy, University of Calabria, Cosenza, Italy

## Abstract

**Background:**

A key role of estrogens in human sperm biology has been recently suggested by aromatase and estrogen receptor detection in human testicular germ cells and ejaculated spermatozoa. However, the involvement of these hormones in the sperm maturation process is still not defined. The aim of this work was to investigate the expression of estrogen receptors, ER-alpha and ER-beta, in human ejaculated immature spermatozoa with excess residual cytoplasm.

**Methods:**

Immunofluorescence labelling has been used to localize ER-alpha and ER-beta proteins in immature spermatozoa isolated by Percoll gradient, while Western blot analysis was carried out on sperm protein extracts.

**Results:**

Both estrogen receptors were localized in excess residual cytoplasm of immature sperm, while sperm tails showed only ER-beta. Furthermore, in the same cells, immunoblots detected the presence of the full-length (~67 kDa) ER-alpha and (~59 kDa) ER-beta proteins, together with a ~50 kDa ER-beta species, lacking in mature sperm.

**Conclusion:**

The present investigation demonstrated ER-alpha and ER-beta presence in excess residual cytoplasm of human abnormal sperm cells, suggesting the hypothesis that both the 'classical' ERs could be able to mediate estrogen action in spermatogenetic cells. Furthermore, the presence of the short ER-beta form in abnormal germ cells and its disappearance in mature sperm, support estrogen modulation via different ER forms during sperm maturation.

## Background

In recent years, a key role of estrogens in differentiation and function of mammalian male germ cells has been suggested by the detection of proteins involved in estrogen biosynthesis and activity. In fact, aromatase and estrogen receptors (ERs) have been revealed in sperm cells at different stages of their maturation process [1-3]. It is known that estrogen action on target cells is mediated by two estrogen receptors, ERα and ERβ, each encoded by a unique gene, differing in the C-terminal ligand-binding domain and in the N-terminal *trans*-activation domain [4]. Different ER variant isoforms have been also identified, but their biological significance is still unknown. Information about the loss of estrogen receptor activity has been provided by the estrogen receptor gene knock out (ERKO) mouse. These animals showed altered sperm count, motility and morphology in the adulthood [5]. Furthermore, a diminuished sperm viability has also been observed in a single case of human inactivating mutation of the ERα gene [6]. These findings suggest the estrogen receptor involvement in the achievement of sperm function.

To date, ERβ appears to be the predominant form of estrogen receptor in developing human germ cells such as spermatogonia, spermatocytes and spermatids [7-9] as only a single report indicated ERα presence in primary human spermatocytes and spermatids [7]. Recently, a differential cell distribution of ERβ splice variants (ERβ2, ERβ4, ERβ5) during spermatogenesis has been demonstrated [10,11]. Furthermore, the total absence of both ERs in seminiferous tubule has been also reported [12]. However, the regulatory role of estrogens during sperm differentiation has not yet been clarified.

Human ejaculate can contain spermatozoa with excess residual cytoplasm which has been retained around the sperm mid-piece due to an incomplete maturation process [13,14]. Previous data from our laboratory [15] have demonstrated aromatase expression in cytoplasmic droplets of immature spermatozoa, indicating a local estrogen biosynthesis. The aim of this study was to provide additional data on estrogen involvement in sperm differentiation, investigating the presence of estrogen receptors (ERα and ERβ) in human ejaculated spermatozoa with excess residual cytoplasm.

## Materials and methods

### Specimens

Semen samples have been obtained from patients who attended University Centre for Fertility Evaluation and the ethical committee members of the University of Calabria approved the investigation programme.

Standard semen parameters were determined according to the WHO [16]. Particularly, sperm morphology was assessed by the May-Grümwald Giemsa staining, observing a minimum of 200 spermatozoa for each sample under an oil immersion lens (×1000). Selected specimens were 10 ejaculates showing asthenozoospermia and a high proportion (15–25%) of spermatozoa with excess residual cytoplasm (abnormal mid-piece droplet greater than one third of the size of the sperm head). The ejaculates from 10 fertile donors served as the control group.

#### Sperm isolation

Sperm cells were isolated from semen on discontinuous Percoll gradient (40%–70%–90%) by centrifugation at 500 g for 20 minutes. Spermatozoa with excess residual cytoplasm were recovered from the 40%/70% interface, while normal sperm were recovered from the 90% layer.

### Antibodies

Anti-ERα primary antibody was mouse monoclonal F-10 (Santa Cruz Biotechnology, Ca, USA) which recognizes epitope mapping at the C-terminus region of the human native ERα. Anti-ERβ primary antibody was rabbit polyclonal H-150 (Santa Cruz Biotechnology, Ca, USA) which recognizes epitope mapping at the N-terminus regions of human native ERβ. Rabbit polyclonal anti β-actin (Santa Cruz Biotechnology, Ca, USA) was also used as loading control. Fluorescein isothiocyanate (FITC) conjugated IgG (Sigma Aldrich, Milan, Italy), Texas-Red conjugated IgG (Vector Laboratories, INC, Burlingame, CA) and horseradish peroxidase conjugated IgG (Santa Cruz Biotechnology, Ca, USA) were used as secondary antibodies.

### Immunofluorescence assay

Sperm cells, recovered from Percoll gradient, were rinsed three times with 0.5 mM Tris-HCl buffer, pH 7.5 and were allowed to settle onto slides in a humid chamber. The overlying solution was carefully pipetted off and replaced by absolute methanol for 7 minutes at -20°C. After methanol removal, sperm cells were washed in Tris-buffered saline (TBS), containing 0.1% Triton X-100 and were treated for immunocytochemistry. Anti-human ERα IgG (F-10) (1:50) and anti-human ERβ (H-150) (1:100) have been utilized as primary antibodies, while anti-mouse IgG Texas-red conjugated (1:50) and anti-rabbit IgG FITC conjugated (1:50) have been used as secondary antibodies. Sperm cells, incubated without the primary antibodies, were utilized as negative controls. Absorption controls were also performed by using the primary antibodies (F-10 and H-150) preabsorbed with an excess of the related purified antigens (ERα blocking peptide: sc-8002P; ERβ blocking peptide :sc-6820P, Santa Cruz Biotechnology) (5 nm/ml), for 48 h at 4°C. The slides were examined under an epifluorescence microscope (Olympus BX41) with a suitable filter for FITC and Texas-Red, observing a minimum of 200 spermatozoa × slide (100× objective) Fluorescent images were captured on a PM-C35DX camera (Olympus), exposure: 9 sec, and printed on Kodak paper (15 × 10). The images were then acquired by Epson Expression 1680Pro scanner at 300 dpi in RGB at 12 bit, and compiled using Adobe Photoshop 7 (Adobe System Inc.)

### Western blot analysis

After Percoll removal, sperm samples were re-suspended in lysis buffer (62.5 mmol/L Tris-HCl (pH 6.8), 150 mM NaCl, 2% sodium dodecyl-sulphate (SDS), 1% Triton X100, 10 % glycerol, 1 mM phenylmethylsulfonylfluoride, 0.2 mM Na_3_VO_4_, 1% aprotinin). Lysates were quantified using Bradford protein assay reagent [17]. Equal amounts of protein (20 μg) were boiled for 5 minutes, separated under denaturing conditions, by SDS-PAGE on 10% polyacrylamide Tris-glycine gels, and then electroblotted to nitrocellulose membrane. Non-specific sites were blocked with 5% non fat dry milk in 0.2% Tween-20 in Tris-buffered saline (TBS-T) for 1 hour at room temperature and incubated overnight with anti-human ERα (F-10, 1:500 dilution), anti-human ERβ (H-150, 1:1000 dilution) and anti-human β-actin. Then antigen-antibody complexes were detected by incubation of the membranes with the appropriate secondary antibodies (anti-mouse or anti-rabbit horseradish peroxidase-conjugated, Amersham, USA) for 1 h at 22°C. The bound secondary antibodies were detected with the ECL Plus Western blotting detection system (Amersham, USA) according to the manufacturer's instructions. Each membrane was exposed to the film for 2 minutes.

Protein extracts from MCF7 (breast cancer cell line) and LnCap (prostate cancer cell line) were cultured as previously reported [18] and used as positive controls for ERα and ERβ respectively. Negative controls were prepared using sperm lysates where antigens were previously removed by pre-incubation with specific antibodies (1 h at room temperature) and subsequently immunoprecipitated with protein A/G-agarose.

## Results

### ERα/ERβ immunofluorescence

**Figure 1:**
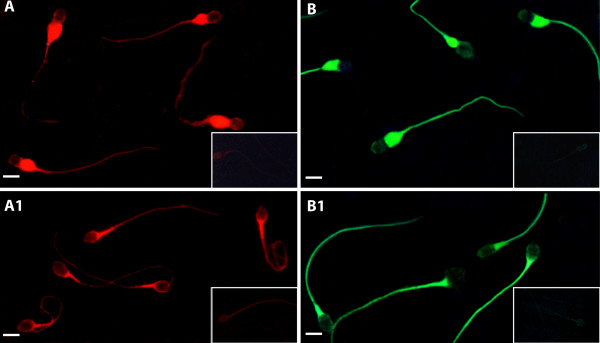
ERα and ERβ immunofluorescence labelling of morphologically normal spermatozoa and spermatozoa carrying superfluous cytoplasm A: ERα red brilliant light (Texas-Red) in excess residual cytoplasm of immature spermatozoa. A1: ERα fluorescence in mid-piece regions of normal sperm. B: ERβ green intense light (FITC) in excess residual cytoplasm and tails of immature spermatozoa. B1: ERβ fluorescence in mid-piece regions and tails of normal sperm. Scale bars 5 μm.

A red brilliant light revealed ERα presence in excess residual cytoplasm of immature spermatozoa, while their heads and tails were not labelled (Fig 1:A). Normal sperm showed ERα only in the mid-piece region (Fig 1:A1).

A green brilliant light detected ERβ in excess residual cytoplasm and in all the tails of immature sperm, but not in their heads (Fig 1:B). Normal spermatozoa revealed ERβ fluorescence in all the tail regions (Fig 1:B1).

The immunostaining specificity was verified by the absence of immunoreaction in negative controls (data not shown) as well as in absorption controls (Fig 1, inserts) The immunofluorescence experiments were repeated 10 times with similar results.

### Immunoblotting

**Figure 2:**
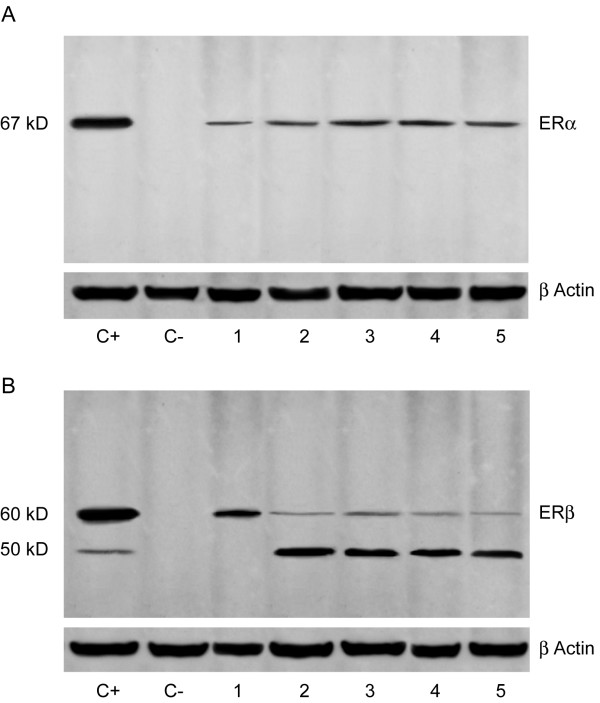
Western blotting analysis of ERα and ERβ in human immature and mature ejaculated spermatozoa. A. Immunoblots of sperm extracts by using anti-ERα: a 67 kDa band has been detected in positive control (MCF-7 extract) (lane C+), normal sperm (lane 1) and immature spermatozoa (lines 2–5); the band was lacking in negative control (lane C-). B. Immunoblots of sperm extracts by using anti-ERβ: two bands, 59 and 50 kDa, have been revealed in positive control (LNCaP extract) (lane C+) and immature spermatozoa (lanes 2–5), while normal sperm have shown only the 59 kDa band (lane 1); both the bands were absent in negative control (lane C-). β-actin (in A and B) serves as a loading control.

The anti-ERα antibody detected a single band corresponding to the molecular weight values of ~67 kDa in normal (Fig. 2:A, lane 1) and immature spermatozoa (Fig. 2:A, lanes 2–5), with a major band in the latter. This band co-migrated with positive control band (MCF7) (Fig 2:A, lanes C+) while no band has been observed in negative control (Fig 2:A, lane C-).

The anti-ERβ antibody (Fig 2:B) showed only the expected band at ~59 kDa in normal sperm (Fig 2:B, lane 1), while two bands were revealed in immature spermatozoa: a weaker one at about ~59 kDa, consistent with the full length ERβ size, and a thicker band at ~50 kDa (Fig 2:B, lanes 2–5). Positive control (LnCap) showed both the ERβ bands (Fig 2:B, lane C+) while no band has been observed in negative control (Fig 2:B, lane C-).

The same results have been obtained in all the 10 samples. The figures 2A and 2B show 4 representative specimens.

## Discussion

In the last stage of mammalian spermiogenesis, the bulk of spermatid cytoplasm is extruded in tubular lumen while a small cytoplasmic mass is retained around the sperm mid-piece as cytoplasmic droplet. This droplet moves to the end of the tail and finally sheds from mature spermatozoa [13]. This process occurs during epididymal transit in non-human species [19-21], but shortly before spermatozoa enter epididymis in humans [22]. Small cytoplasmic droplets are often observed in rapidly fixed preparations of human ejaculated sperm, but they are not considered deleterious to sperm functional properties [13,14].

However, human semen can also contain abnormal spermatozoa with large amounts of excess cytoplasm adhering to the mid-piece, due to an arrest of spermatid differentiation at a late stage of spermiogenesis [13,14]. A high incidence of excess residual cytoplasm bearing spermatozoa has been observed in semen from smokers [23] and men with varicocele [24] and defective sperm functions [25,26]. Retention of excess residual cytoplasm has been associated with oxidative stress as a consequence of enhanced reactive oxygen species production [27-30].

This is the first report identifying estrogen receptors, ERα and ERβ, in excess residual cytoplasm of human ejaculated immature spermatozoa, consistent with our previous data [15] which showed the presence of aromatase in the same cell site. Aromatase is the enzyme responsible of the conversion of androgens to estrogens, therefore, our past and present data suggest that cytoplasm of testicular germ cells contains a local estrogen source and also its own receptors for a possible autocrine estrogen regulation of spermatid differentiation in the testis.

The expression of estrogen receptors in human sperm cells is still a matter of debate. ERβ is the estrogen receptor isoform detected in testicular germ cells by many authors, while the presence of ERα in the same cells is widely controversial [7-9]. However, the expression of both ERs has been recently reported in mature spermatozoa [31-33].

It is generally accepted that residual cytoplasm of immature sperm contains discarded components of the original spermatids; therefore our results suggest that not only ERβ but also ERα could mediate estrogen action in spermatogenetic cells. According to this, a recent report demonstrated the presence of both estrogen receptor isoforms in round immature sperm cells of human ejaculate [32]. However, sperm cells with excess residual cytoplasm interact with epididymal fluid and fluids produced by accessory sex glands during their transit in reproductive ducts, as witnessed by their acquired motility, a property linked to the post-testicular sperm maturation. Therefore, it might be taken in account that these interactions could also influence the expression of sperm signalling proteins.

Spermatozoa with excess residual cytoplasm, in addition to the full-length ERβ isoform, showed also the expression of a ~50 kDa ERβ form, which was lacking in mature sperm. A 50 kDa ERβ, indicated as ERβ short form, has been recently described in round germ cells of human ejaculate [32] and a ERβ short form has been detected in human testis [9,10]. Therefore, it is reasonable to hypothesize that the ERβ variant of immature spermatozoa could correspond to the ERβ short form.

This interesting finding supports the hypothesis that estrogen could modulate their action through different ER forms during the sperm maturation process. Furthermore, ERβ immunolocalization in the tails gives the cue to further studies to verify if estrogen can regulate sperm motility in these ejaculated immature forms, as in normal sperm [31].

Regarding mature spermatozoa, the present investigation has confirmed our previous data [31] identifying only the full-length isoforms of the two ERs, particularly ERα in mid-piece region and ERβ in all the sperm tail. There are some discrepancies with a recent report showing ERα in the head of the mature sperm [33] but this could happen mainly from the use of different anti- ERα primary antibodies.

## Conclusion

The present investigation demonstrated ERα and ERβ presence in excess residual cytoplasm of human immature sperm cells, suggesting the hypothesis that both the 'classical' ERs could able to mediate estrogen action in spermatogenetic cells. Furthermore, the presence of the short ERβ form in immature germ cells and its disappearance in mature sperm, support estrogen modulation via different ER forms during sperm maturation.

## Authors' contributions

**RV: **the author responsible for performing the immunohistochemical expriments and participating in the analysis and interpretation of data.

**SL: **the author responsible for sperm isolation and protein extraction

**AS: **the author responsible for performing Western blot analysis

**CA: **the author responsible for conception, design, analysis and interpretation of data as well as of drafting manuscript
